# Collagen/Chitosan Functionalization of Complex 3D Structures Fabricated by Laser Direct Writing via Two-Photon Polymerization for Enhanced Osteogenesis

**DOI:** 10.3390/ijms21176426

**Published:** 2020-09-03

**Authors:** Irina Alexandra Păun, Cosmin Cătălin Mustăciosu, Roxana Cristina Popescu, Bogdan Ştefăniţă Călin, Mona Mihăilescu

**Affiliations:** 1 Center for Advanced Laser Technologies (CETAL), National Institute for Laser, Plasma and Radiation Physics, RO-077125 Magurele, Romania; bogdan.calin@inflpr.ro; 2 Faculty of Applied Sciences, University Politehnica of Bucharest, Splaiul Independentei 313, RO-060042 Bucharest, Romania; mona_m@physics.pub.ro; 3 Department of Life and Environmental Physics, Horia Hulubei National Institute for Physics and Nuclear Engineering, Reactorului 30, RO-077125 Magurele, Romania; cosmin@nipne.ro (C.C.M.); roxpopescu@yahoo.co.uk (R.C.P.); 4 Department of Science and Engineering of Oxide Materials and Nanomaterials, Politehnica University of Bucharest, Gheorghe Polizu 1-7, RO-011061 Bucharest, Romania

**Keywords:** laser direct writing, 3D structure, collagen/chitosan functionalization, osteogenesis

## Abstract

The fabrication of 3D microstructures is under continuous development for engineering bone substitutes. Collagen/chitosan (Col/CT) blends emerge as biomaterials that meet the mechanical and biological requirements associated with bone tissue. In this work, we optimize the osteogenic effect of 3D microstructures by their functionalization with Col/CT blends with different blending ratios. The structures were fabricated by laser direct writing via two-photons polymerization of IP-L780 photopolymer. They comprised of hexagonal and ellipsoidal units 80 µm in length, 40 µm in width and 14 µm height, separated by 20 µm pillars. Structures’ functionalization was achieved via dip coating in Col/CT blends with specific blending ratios. The osteogenic role of Col/CT functionalization of the 3D structures was confirmed by biological assays concerning the expression of alkaline phosphatase (ALP) and osteocalcin secretion as osteogenic markers and Alizarin Red (AR) as dye for mineral deposits in osteoblast-like cells seeded on the structures. The structures having ellipsoidal units showed the best results, but the trends were similar for both ellipsoidal and hexagonal units. The strongest osteogenic effect was obtained for Col/CT blending ratio of 20/80, as demonstrated by the highest ALP activity, osteocalcin secretion and AR staining intensity in the seeded cells compared to all the other samples.

## 1. Introduction

An important strategy in tissue engineering relies on seeding and growing cells on porous 3D structures that support in vitro tissue formation and maturation, followed by tissue implantation of the cell-seeded scaffolds into the patient for further cell growth [1].

Common methods of fabrication of such 3D structures include solvent casting with particulate leaching [2], freeze-drying [3], gas foaming [4], electrospinning [5] and phase separation [6]. Lately, more advanced technologies such as three-dimensional (3D) printing, also known as additive manufacturing or rapid prototyping, emerged as suitable for tissue engineering [7]. In this case, structure fabrication is guided by a computer model in a layer-by-layer fashion, with high structural complexity [8]. Among these methods, laser direct writing via two-photons polymerization (LDW via TPP) offers the advantage of high spatial resolution i.e., below the diffraction limit, full reproducibility of the structures and the possibility to imprint any desired geometries [9,10,11].

IP type photoresists are liquid and exposure is realized using laser direct writing techniques. Exposure initiates a chain polymerization process, which results in solid structures. The structures’ developing is made for washing away the non-polymerized material i.e., the remaining monomers, through immersion in an appropriate solvent. Fluid movements, surface tension of the evaporating solvent and polymer shrinkage can affect the resulting structures. Therefore, a high degree of polymerization is required for the structures to retain their shape and size after development. In our experiments, we studied the effects of both the geometry and functionalization. Since the functionalization process comes after the laser writing process, we must first obtain microstructures with good structural integrity that can withstand the dip-coating process and maintain their geometrical features. Besides all these properties, for biological application the polymerized material must also be biocompatible.

LDW via TPP stands out from the current 3D printing techniques and relies on focusing a femtosecond laser beam in a photoreactive polymer, where it triggers a highly localized photomodification [10]. LDW via TPP has unique advantages among other printing technologies, such as spatial resolution below the diffraction limit, no limitations concerning the 3D architectures that can be obtained and high reproducibility of the 3D structures [7,8,9,10]. For tissue engineering, LDW via TPP provides the means to fabricate 3D structures that sustain the healing of damaged tissue [9,10,12,13,14]. In this regard, a lot of attention is dedicated to the optimum choice of the structural architecture and of the most suitable polymer. Beside its suitability for two-photon absorption at the laser wavelength, the photoreactive polymer also has to be biocompatible [ref 10 from the original manuscript]. LDW via TPP technique is not very commonly used for biological applications because of the toxicity of many of the available photopolymers; extensive post-fabrication washing is generally used when processing such materials [15]. The most suitable photopolymer for biological applications should have a high degree of conversion from monomer to polymer in order to decrease the amount of residual monomer and should possess a fast polymerization kinetics. Furthermore, it would be mostly desirable that the photopolymer has mechanical and even degradation properties adapted for each tissue engineering application [8,9,10,15]. Despite these difficulties, there is an ongoing progress in developing and testing various photopolymers for LDW via TPP processing for tissue engineering. For example, dental applications successfully employed methacrylates, while, for the regeneration of soft tissue, biocompatible photopolymers have been synthesized from biological polymers such as chitosan and gelatin [15]. Moreover, the 3D printer manufacturers such as Nanoscribe (which is the system we used for structures’ fabrication) have developed dedicated photopolymers optimized for being processed with the Nansocribe technology. Among these, IP-L photpolymers have been shown to have high biocompatibility in vitro for bone-forming cells, with promising results for bone regeneration in vivo [13,14].

IP-L780 is a commercially available photopolymer developed by Nanoscribe GmbH. It is a biocompatible liquid formulation with optimized sensitivity for fast 3D structuring ref. [13]. We employed IPL-780 for the present work because the producer recommends it for tissue engineering applications and because it has proven good ability to produce structures sized down to 150 nm, with low stress, little shrinkage and good adhesion on glass substrates [13]. Moreover, the biocompatibility of the IP-L780 structures produced using LDW by TPP and their potential for bone tissue regeneration was recently tested by us osteoblast-like cells cultures [13,14].

A solution to this problem is the post-printing surface functionalization that preserves the bulk properties of the 3D structures, such as the structural rigidity required for bone regeneration [12,13,14]. Despite the scientific interest, alternative approaches for the functionalization of 3D-printed structures are limited. For example, a recent work investigated the ability of initiated chemical vapor deposition (iCVD) process to coat 3D-printed shapes composed of poly(lactic acid) and acrylonitrile butadiene styrene with hydrophilic (poly(1H,1H,2H,2H-perfluorodecyl acrylate) (PPFDA) and hydrophilic (poly((2-hydroxyethyl methacrylate)-co-(ethylene glycol diacrylate)) (P(HEMA-co-EGDA)) polymers [12]. The approach has several major disadvantages, some being directly related to tissue engineering applications. One limitation is that iCVD method and the subsequent coating procedure involve complicated equipment and time-consuming, multi-step experimental procedures. Also, the 3D-printed structures reached a temperature of 80 °C and the stage temperatures used for post-processing coating went up to 45 °C. Knowing that Col and CT, individually or in blends, degrade at temperatures between 35–50 °C refs. [16,17,18,19,20,21,22,23], such an approach would certainly destroy the Col/CT blends used for the functionalization of our structures. Another disadvantage is that thermally insulating properties of 3D-printed plastics represent a challenge for iCVD process due to large thermal gradients along the structures during processing that further affect the uniformity of the coatings. Solid free form-based 3D polycaprolactone/poly(lactic-co-glycolic acid) scaffolds were successfully coated with recombinant mussel adhesive proteins MAPs by simply dipping the scaffolds into MAP solution [24]. Although the scaffolds’ functionalization with MAPs enhanced the attachment, proliferation, and osteogenic differentiation of human adipose tissue-derived stem cells, the achievable 3D geometries that could be printed for the scaffold’s architecture were limited and the homogeneity of the coating was not demonstrated. An UV-curable resin with an embedded alkyl bromide initiator for atom transfer radical polymerization was used for modifying the surfaces of 3D structures fabricated via stereolithography [25]. However, the experimental procedures were complex and did not apply to biocompatible polymers such as the ones used in our study. In addition, the method was capable of fabricating metallic structures, which are less suitable for tissue engineering than the biopolymers we used for functionalizing our structures. In this context, the functionalization of 3D polymeric structures with biologically active molecules for tissue engineering remains challenging.

Until now, a variety of materials and their combinations have been used for bone tissue engineering [16]. Natural materials such as chitosan (CT) and collagen (Col) bring significant advantages since they are nontoxic and support cell attachment and proliferation [17,18]. CT is used for bone tissue engineering due to its optimum strength, degradation resistance, and cell-supportive properties [19]. In vitro studies have demonstrated that CT promotes the adhesion and proliferation of osteogenic cells and mesenchymal stem cells [20]. Col is another material with great potential for bone tissue engineering. For example, bone marrow cells grown on Col were able to differentiate into osteoblasts, as evidenced by increased osteogenic gene expression and elevated alkaline phosphatase activity [21]. However, both CT and Col are difficult to process, having poor mechanical properties and sometimes they might not be suitable for managing infected sites [22]. Finding the best way to functionalize the surface of 3D printed structures with these materials for bone tissue engineering applications remains a challenge.

In a recent work, we describe the fabrication by LDW via TPP of complex 3D structures, with micrometric spatial resolution, having hexagonal and ellipsoidal unitary elements separated by cylindrical pillars [14]. In this work, we functionalized these structures by dip coating in Col/CT blends. Our aim was to enhance the osteogenesis in osteoblast-like cells seeded on the functionalized 3D structures.

By the proposed approach, we combine some of the unique advantages of LDW via TPP technique that make it useful for systematic studies (such as ability to produce 3D structures with no constraints and full reproducibility for the obtained geometries), with surface functionalization of the structures with optimized combinations of materials such as Col and CT. In particular, we addressed the influence of the Col/CT blending ratios over the 3D structures in terms of biocompatibility and osteogenic potential of osteoblast cells seeded on them. Structure morphology, wettability and biocompatibility was investigated, along with the expression of specific osteogenic markers such as ALP activity, osteocalcin secretion and Alizarin Red staining (ARS) as dye for mineral deposits. The experiments were performed systematically as a function of Col/CT blending ratio that covered the 3D structures. Based on the experimental findings, the Col/CT blending ratio that best enhanced the osteogenesis was found.

## 2. Results and Discussion

### 2.1. Design, Fabrication and Characterization of Functionalized 3D Structures

**Figure 1:**
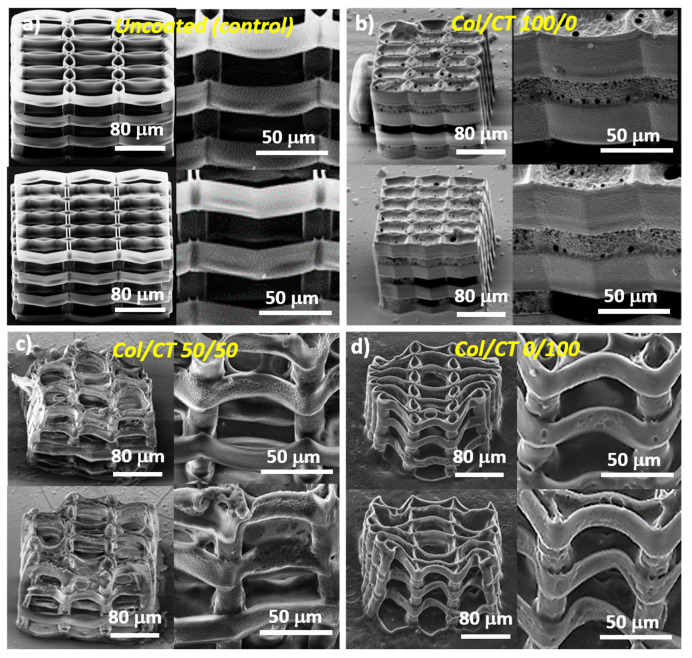
SEM micrographs of 3D structures produced by LDW via TPP: (**a**) uncoated structures; (**b**–**d**) 3D structures functionalized with Col/CT with different blending ratios. For (**a**–**d**): the upper panels illustrate 3D structures with ellipsoidal units; the lower panels illustrate 3D structures with hexagonal units; the left panels show overviews of the structures tilted with 30 deg; the right panels show closer views of the structures depicted in the left panels.

The starting point is represented by the optimized microstructures obtained in our previous studies [14]. These structures were fabricated by LDW via TPP of IP-L780 photopolymer, using a specific design and fabrication procedure that promoted the cell attachment and interconnections, while retaining good structural integrity. Elements of the microstructures were either elliptical or hexagonal having a length of 80 µm, width of 40 µm, about 14 µm tall, separated by 20 µm cylindrical pillars, placed in the overlapping areas (Figure 1a).

The 3D structures were fabricated by two-photon polymerization, where the size of the volume pixel (voxel) is 2 µm width and 4 µm height [14]. However, neighboring lines were not as tall as the voxel itself, as we imposed a 2 µm overlap to provide good structural integrity and polymerization degree.

Following dip coating of these 3D structures in Col/CT blends, the backbone architecture of the laser-imprinted structures changed drastically. In addition, the nature of these changes was found to depend on the blending ratios between Col and CT (Figure 1b–d). Higher amounts of Col in the blend did not change the architecture of the 3D structure underneath; in this case, we found a continuous sheet that covered and “sealed” the whole structure and having several micrometric pores (as evidenced by the insets from Figure 1b). On the other hand, the geometry of the structure was affected quite strongly when the amount of CT in the blend was increased. Ellipsoidal and hexagonal units were significantly deformed, while the vertical pillars remained almost unchanged (Figure 1c). Moreover, remaining of the continuous sheet (similar with that observed in the case of pure Col) sealed some parts of the 3D structure underneath (as shown by the insets from Figure 1c). With increasing CT content in the blend, the structure deformation was less dramatic and the free spaces between the unitary elements were no longer sealed by a coating sheet (as evidenced in Figure 1d).

The optimized structures design was determined in previous experiments [14]. The layers are 14 µm tall and consecutive layers are 16 µm apart. The ellipses are 80 µm long and 40 µm wide, along the major and minor axes. Wall thickness is determined by the size of the voxel itself, which is 2 µm in this case. Col/CT functionalization determined strong deformation of the structures. For only Col functionalization, the structures are enclosed in a layer of collagen, and therefore rendering nonexistent structures porosity, from a practical point of view. Col/CT blends do not enclose the structures, but in turn determine strong deformations. As a result, porosity is difficult to measure and strongly varies for each sample. SEM micrographs do not indicate a noticeable increase in wall thickness.

The shrinkage is a general problem for the fabrication of complex micro/nanostructures and is mainly caused by the material densification as compared to the material before polymerization that results in volume reduction; the geometrical deformations of 3D printed structures mostly appear because of the surface tension effects that occur during the developing and drying processing steps [26], which we described in Materials and Methods section. In our experimental conditions, the developer (PGMEA) was especially designed for IP-L780 polymeric structures fabricated using Nanoscribe technology and therefore its evaporation during sample developing process does not cause the shrinkage of the 3D structures. This idea is sustained be the fact that in Figure 1a the uncoated 3D structure has a sharp architecture, with no signs of shrinkage. In contrast, structure deformation and especially shrinkage along the long axis of the ellipsoidal elements occurred for Col/CT functionalized structures (Figure 1c,d). The shrinkage becomes stronger with increasing CT content in the blend (Figure 1c,d). Since both Col and CT were dissolved in acetic acid, the solvent is certainly one reason causing the structures deformation. However, the fact that the structures functionalized with only Col were not deformed (Figure 1b) means that the acetic acid is not the only factor responsible for structures’ deformation. Instead, we suspect that the combinations of Col/CT with a certain amount of acetic acid deformed the structures. As we stated before, it seems that CT was more influent than Col in this regard. An important aspect is that this deformation did not interfere with the cellular attachment. On the opposite, it seems that the design of the structures allowed for good volumetric cell migration, not only due to the free spaces between neighboring elements, but also due to the flexibility of the structures’ walls. More precisely, while the structures showed good mechanical resistance in the up and down direction, they are prone to side-to-side movements (Figure 1c,d). This is advantageous for cell migration (Figure 1d), but it also makes the scaffold prone to deformations determined by fluid movements and surface tension of any evaporating liquid, such the acetic acid used for preparing the Col/CT blends for structures functionalization. Moreover, structure deformation due to shrinkage may occur following the dipping procedure in the Col/CT solutions used for structures functionalization. Further investigations are required to determine and quantify the set of factors that result in structure deformation.

A major point is related to the chemical composition of the Col/CT coating that may account, at least to a certain extent, for samples’ wettability. In this regard, we argue that the dip coating technique used for the functionalization our structures is generally recognized as suitable for coating complex shapes and curved parts (like those in the 3D geometries presented in this study) that could not be coated by any technique [27]. Moreover, the chemical composition of other complex structures functionalized by dip coating in Col and CT has been extensively investigated by Fourier transformed infrared spectroscopy, for both individual materials as well as in blends, and therefore the results could be easily extrapolated to our experimental conditions [28]. In our case, the optical inspection provided by the detailed SEM analysis provide evidence on the degree of uniformity of the coating and of other particular aspects regarding the role of Col/CT blending ratio on the uniformity of the coating of the 3D structures and that were discussed in this section.

**Figure 2:**
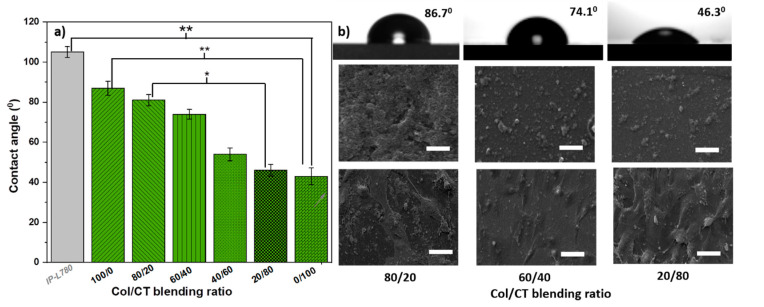
(**a**) Contact angles measured on IP-L780 photopolymer flat surfaces and on Col/CT with different blending ratios drop casted on photopolymer flat surfaces. Each bar represents the mean ± STD. Statistical significance determined by Student’s *t*-test (* *p* ≤ 0.05; ** *p* ≤ 0.001). (**b**) (upper panel) Images of liquid drops and contact angles on surfaces with the indicated Col/CT blending ratios; (middle panel) SEM micrographs of the corresponding surfaces; (lower panel) SEM micrographs of cells seeded on the surfaces. The scale bars in the SEM images correspond to 50 µm.

To avoid any influence of the structure geometry on the wettability of the samples, we measured the water contact angles on flat surface made by drop casting of the same materials as used for the functionalized structures. As such, water contact angles measured on Col/CT blends casted on flat photopolymer surfaces are depicted in Figure 2a. Figure 2b shows water drops on Col/CT blends with different blending ratios; the morphology of these surfaces and the seeded cells are also shown. The mean ± standard deviation of the contact angle measured on the material used for building the backbone (IP-L780 polymerized in the form of a flat surface) was of 105° ± 3°, indicating that the hydrophobic character of the backbone 3D structure. This is in fact a major problem of 3D printed structures for bone tissue engineering that generally have hydrophobic surfaces that impede the cellular attachment and differentiation. This was also our case, since the uncoated photopolymer which was in fact the backbone material of the 3D structures had the water contact angle above 90°.

The contact angles measured on Col/CT surfaces casted on flat photopolymer substrates were statistically lower than on the photopolymer surface, proving that the functionalization of the photopolymer with Col/CT increased the hydrophilicity of the samples. Moreover, the contact angles decreased with decreasing Col and increasing CT contents in the blend. Specifically, for pure Col surfaces the contact angle was of 87° ± 4°and continued to decrease with increasing CT content in the blend until reaching 43° ± 3° for pure CT surface. These results are in fair agreement with previously reported values. It has been shown that the water contact angle on Col substrates varies between 83° and 87° [23]. It is also know that CT has a hydrophilic nature that supports the attachment and proliferation of bone-forming osteoblast cells as well as formation of mineralized bone matrix in vitro [20].

In our experimental conditions although both CT and Col are hydrophilic but with quite high contact angles i.e., above 70°, the hydrophilic character of the Col/CT blends was more pronounced than each of the individual Col and CT components reported by the above cited literature. Most likely that also the morphology of the Col/CT surfaces played an important role in what concerns the wettability. As evidenced by Figure 2, the contact angle for the Col/CT surfaces decreased with increasing CT content in the blends, while the surface becomes smoother.

**Figure 3:**
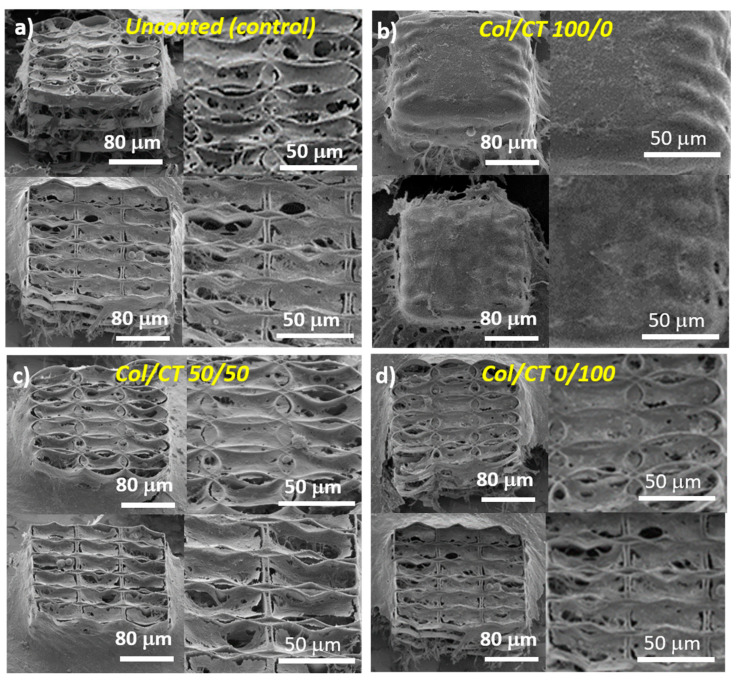
SEM micrographs of MG-63 osteoblast-like cells seeded for 3 days on: (**a**) uncoated 3D structures; (**b**–**d**): 3D structures functionalized with Col/CT with different blending ratios. For (**a**–**d**): the upper panels illustrate cells seeded on structures with ellipsoidal units; the lower panels illustrate cells seeded on structures with hexagonal units; the left panels show overviews of cell-seeded structures tilted with 30°; the right panels show closer views of cell-seeded structures depicted in the left panels.

Moreover, the cells attachment also increased with CT content in the blends; likely it was promoted by the increase of surface hydrophilicity, which in turn corresponded to an increase in surface smoothing. The influence of surface wettability has been intensively studied in the context of favouring the cell attachment [29,30,31]. A hydrophilic surface is characterized by the fact that the forces of adhesion overcome the forces of cohesion at the surface of the material, thus supporting the attachment of cells. Previous studies reported that CT surfaces increase the protein adsorption, the cell attachment and act in the benefit of osseointegration [32]. In our case, a similar effect of cell attachment promotion was noticed in case of high content CT in blend (Figure 3b–d).

The reason for which we presented the wettability measurements of flat Col/CT surfaces is because the height of the ellipses and hexagons within the 3D structures goes up to 25 μm and the thickness (related to the minimum volume of polymerized material named voxel [10,11,12,13]) of few micrometers. These dimensions, much smaller than any water droplet, make impossible to actually measure the wettability directly on the 3D structures. Another reason is that any droplet of liquid used for contact angle measurements would be much higher in diameter that the lateral size of our structures which goes up to 200 μm (as observed from the scales in Figure 1). An additional limiting factor for measuring the water contact angle directly on the 3D structures is that the complexity of the structures triggers the formation of large air pockets under the water droplet, therefore the contact angle measurement on such complex structures would not really reflect the real wettability of the structures’ walls on which the cells actually adhered. To sustain the validity of the contact angle measurements on flat surfaces made of Col/CT blends, we performed preliminary in vitro studies regarding the cells’ adhesion by fluorescence microscopy visual inspection. As it can be observed in the Supplementary Material, the cells attachment on the flat Col/CT surfaces follows the same trend as the cells attached on the 3D structures illustrated in Figure 3. Specifically, for both flat Col/CT surfaces and 3D structures functionalized with similar Col/CT blends, the cellular attachment increased with increasing CT content in the blends. This finding, along with the generally accepted hypothesis that the cellular attachment directly relates to substrate wettability, provides a reasonably strong base to correlate the wettability trends observed for the flat Col/CT surfaces to the wettability of the 3D structures functionalized with similar Col/CT blending ratios.

It was found that the biological performances of the photopolymerizable materials were limited by the characteristics of the photopolymers employed for the laser writing process, which were generally biocompatible, but did not possess specific properties (such as the water affinity, surface roughness, the ability to be modeled, biodegradability, elasticity or stiffness) that sustain cell growth and differentiation into functional tissues [9,10,11]. As emphasized by the contact angle measurements (Figure 2a), the IP-L780 photopolymer used in this study has a strong hydrophobic character, generally causing a rejection of water-based liquids such as the cell culture medium, thus affecting the interaction between the cells and the surface of the structure. The coating with Col/CT blends aims to improve the wettability of the IP-L780.

### 2.2. Biological Assessments of Functionalized 3D Structures

The morphological investigations of MG-63 osteoblast-like cells cultured on Col/CT functionalized structures are shown in Figure 3. The cells were able to attach and grow on all types of structures. However, some differences in shape and density were noticed. In the case of uncoated structure with hexagonal units, numerous attachment points were available for the cells to bind to, resulting in a dense and interconnected network after three days in culture (Figure 3a lower panel). On the other hand, in the case of the uncoated ellipsoidal 3D structure (Figure 3a upper panel), the aspect of the cells network was slightly less dense. Probably due to less attachment points available, but favored by the dimension of the component units, the cells positioned themselves in the round units of the 3D structures, showing a stretched morphology.

Structures functionalization changed the cells behavior. An increase in cells density was clearly evidenced for Col/CT 50/50 (Figure 3c) and Col/CT 0/100 (Figure 3d) as compared to the non-functionalized (uncoated) structures.

On the Col/CT 100/0 samples, the cells formed a thick continuous monolayer at the surface of the 3D structures, which suggested that the cells were not able to bind to the morphological elements of the structures and rather cohered to each other, forming a dense network covering the whole structures.

This phenomenon can be explained by properties of the coating material such as its hydrophobic character (Figure 2), viscosity of the coating solution, but also by the morphology of the 3D structures, namely the porosity (Figure 1). Figure 1b shows that the blend made of 100/0 Col/CT sealed the entire surface of the 3D structure, disabling many of its binding points that could have helped the cells attachment. While the viscosity of the Col/CT solution might have been too high and added to the hydrophobicity of the resulting coating, the introduction in vacuum of the collagen dip-coated structure we would only have unblocked the pores of the structures, whereas the inner walls of the structures would remain uncoated. Thus, although we would have facilitated the access of the cells inside the structure, the interaction with the inner walls would be the same as in case of the uncoated structures. The final result would then be partially false. Further studies on quantitative evaluation of structures’ porosity and viscosity of the dip coating solution would be helpful for better understanding the behavior of these samples and will be the subject of future experiments.

We chose this particular time interval of three days of cell culture for having an optimum cell number density attached on the 3D structures. The cellular behavior observed at shorter time intervals would not be reliable because there would be too few cells attached on the structures. On the other side, longer time intervals were not used for SEM analysis for avoiding too numerous cells that overlap on and within the 3D structures, since the cells divide and grow continuously; a large number of cells growing on and inside the 3D structures would impede detailed observations about the morphology and attachment of individual cells on the structures.

The cell culture density was controlled by using the same cell number/cell medium volume ratio for all samples. Moreover, the same cell number was diluted in the same volume. The attachment time of cells was one hour and afterwards more culture medium was added. This protocol was applied for all samples. The porosity of the structures is the same for all samples in each morphological category, as we used the same 3D architectures for all structures in the same group (ellipsoid respectively hexagonal units). The coating (Col/CT at different ratios) changed the surface (the walls) character of these structures and thus altering their response to cell culture medium. In our previous study [14] we proved that cells were able to penetrate these structures, due to the dimension of its architectural elements. Depending of their morphology (ellipsoid or hexagonal), they enabled different attachment points for the cells, thus affecting the way the cells interacted with the structures. Here, the interaction between cells and the structures was altered by the wettability of the coated surface, rather than its porosity. In Figure 3b is represented the Col/CT 100/0 sample, which was characterized by a strong hydrophobic character. As observed in Figure 3b, the coating covered the exterior walls of the scaffold, sealing its pores. Thus, in this case, the cells laid on the coating and were not able to penetrate the interior of the structures.

The two architectures of the structures we used in this study, namely ellipsoidal and hexagonal, have also been described in a previous work [14] where we proved to be a close relationship between the morphology of the substrates and the cell development. The structures enable different attachment points depending on their architecture. The hexagonal structure enables more attachment points for the cells whereas cells showed a fragmented morphology. On the other hand, the ellipsoidal structure enables a circular shape of the attached cells, in both cases the cells penetrating the interior of the structures.

The weak ability of the cells to bind to a hydrophobic material has been ascribed to an entrapment of air bubbles in these samples due to hydrocarbon contamination, which interferes with the protein adsorption and the cell-receptors [32,33].

Our experimental findings integrate well into the broader scientific context of wettability-cell behavior. It has been shown that both wettability and topography of a surface can influence cell adhesion and spreading: cell adhesion increases with surface roughness, while cell spreading ability decreases [29]. In terms of wettability, a medium character was shown to be the most favorable for cells growth, extreme hydrophilicity/hydrophobicity was shown to prevent the cells attachment.

**Figure 4:**
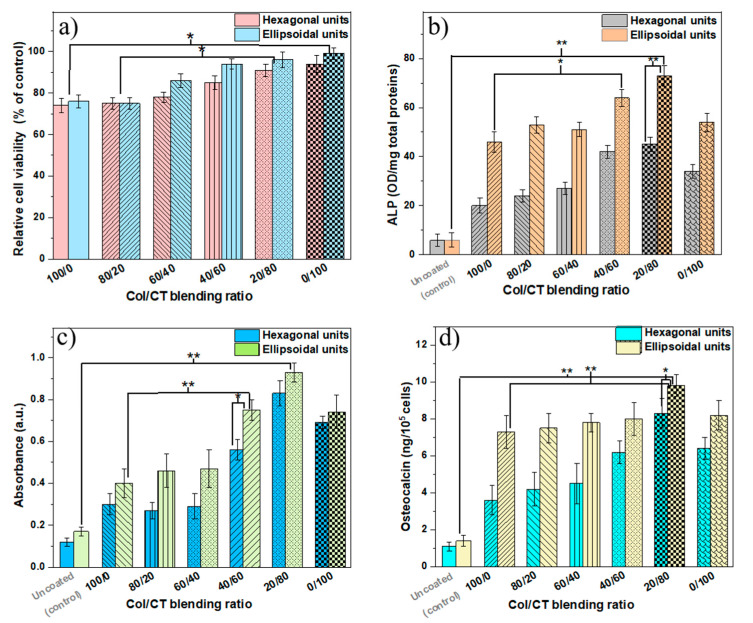
(**a**) Viability of cells seeded on 3D structures functionalized with Col/CT of various blending ratios The viability was measured relative to controls (uncoated) structures; (**b**) ALP activity normalized to protein content for cells seeded on 3D structures functionalized with Col/CT of various blending ratios; (**c**) Absorbance measurements for Alizarin Red marking of mineral deposits from cells seeded on 3D structures functionalized with Col/CT of various blending ratios; (**d**) Absorbance measurements for Osteocalcin secretion from cells seeded on 3D structures functionalized with Col/CT of various blending ratios. Each bar represents the mean ± STD. Statistical significance determined by Student’s test (* *p* ≤ 0.05; ** *p* ≤ 0.001).

Next, the soluble tetrazolium salt viability (MTS) assay was used to investigate the viability of the cells cultured on the Col/CT functionalized structures, as relative to uncoated (control) ones (Figure 4a). Results showed an increase in relative viability with increasing CT content in the blend. Samples with preponderant CT in the composition revealed a biocompatible behavior (viability above 80%, according to [34]). On the opposite, the cells cultured on samples with higher Col content in the blend revealed a decrease in viability, which can be due to the inability of cells to penetrate the structure because of the continuous sheet that sealed the whole structure that also provided less binding points for the cells (as already evidenced in Figure 1b).

The bone tissue forming potential of the Col/CT functionalized 3D structures was evaluated through the cells’ differentiation and mineralization ability after four weeks in culture. This was achieved by measuring the alkaline phosphatase (ALP) and osteocalcin production as biomarkers for these processes along with the alizarin red staining (ARS) assay as direct indicator of mineralized tissue [35,36].

Our investigations showed an increasing ALP production with increasing CT content in coating blends (Figure 4b). The maximum ALP amount was noticed for Col/CT blending ratio of 20/80. Moreover, a significantly higher amount of ALP was measured in case of 3D structures with ellipsoidal units compared to those with hexagonal units. By extrapolating these results to the morphological investigations discussed above, the increase of ALP production in the cells seeded on 3D structures with ellipsoidal unit is correlated with the elongated morphology of the cells (as evidenced in Figure 2c,d upper panels). Thus, it appeared that the morphology of the ellipsoidal units favored the early differentiation of osteoblast-like cells.

Similar observations were made for osteocalcin and alizarin red staining (ARS) measurements (Figure 4c,d). Osteocalcin is a late biomarker for osteoblast cells differentiation and its presence is directly proportional to the deposited mineral [36,37,38]. On the other hand, ARS binds to the mineral depositions in the cell cultures and is a direct indicator of the quantity of minerals in the samples [36]. Structures with ellipsoidal units showed better results in terms of ALP production than the structures with hexagonal units. Again, for both types of structures, the best results were obtained for 3D structures functionalized with Col/CT blends of 20/80 blending ratio, which resulted in highest osteocalcin secretion and ARS intensity. Furthermore, for 3D structures with ellipsoidal units the osteocalcin secretion and ARS intensities were higher than for the structures having hexagonal units. Similar to the experimental data concerning the ALP production, this result can be explained by means of the elongated cells morphology observed in the case of these samples.

As shown in our previous study [13], the ellipsoidal structures enabled a better behavior of the cells’ attachment, as they followed the architecture of the structure’s walls. Although the hexagonal structure enabled more attachment points, their morphology was rather fragmented, affecting their biological behavior. These could explain the better osteogenic activity of the ellipsoidal structures.

Our results are in good agreement with previous studies on Col and CT effects on the osteogenesis. CT is a naturally occurring polymer with minimal foreign-body response, non-toxic degradation products, high biocompatibility, biodegradability and osteoinduction. CT is particularly attractive for bone regeneration because it promotes the attachment and proliferation of osteoblasts and the formation of mineralized bone matrix in vitro [20]. For example, CT scaffolds exhibited high osteoconductivity in surgically created bone defects [39]. CT accelerated the wound healing and showed antimicrobial properties [40,41]. Nevertheless, CT is mechanically weak, with compressive modulus of pure CT structures significantly lower magnitudes than the cancellous bone [42]. In addition, CT does not have the structural stability and is not able to preserve a specific shape in aqueous environments, which is a critical requirement in the implantation at a bone defect site. In order to improve its mechanical properties and structural integrity when hydrated, CT is sometimes blended with other materials, an interesting candidate for this purpose being Col [43]. Col has been extensively used in the biomedical field mainly because it forms fibers with high strength and stability. In addition, Col is a good surface-active agent, with an important role in the functional expressions of cells leading to the formation of tissues and even organs [44]. Col exhibits biodegradability, weak antigenicity, good biocompatibility and increases the osteogenic gene expression and the alkaline phosphatase activity in bone-forming cells [21,45]. An important aspect for the engineering of bone tissue is that Col is the most abundant extracellular matrix protein of bone and plays a major role for its strength [46]. Combinations of Col/CT fused with glycolic acid were used as bone graft substitute to promote bone tissue regeneration [43]. The results of our work demonstrate that the Col/CT blends are suitable candidates for the functionalization of complex 3D structures for bone tissue engineering and that the structures functionalization can be achieved via a simple i.e., non-chemical dip coating process.

For morphological examinations by SEM, we selected the most representative samples, namely the extremes and middle of the Col/CT blending ratio range. Moreover, SEM is a qualitative investigation, while the other in vitro methods employed in this study have a statistic and quantitative character. A difference of 20% in the concentration of each polymer in the blend was generally used for these quantitative investigations. A difference of only 10% (between 60/40 and 50/50 or 50/50 and 40/60) fits into the 10% interval of error which is generally accepted in biological investigations. In our experimental conditions, an interval equal or below 10% between Col/CT blending ratios is not relevant for the observed trends of the quantitative measurements such as wettability and biological assessments nor for qualitative morphological investigations.

## 3. Materials and Methods

**Figure 5:**
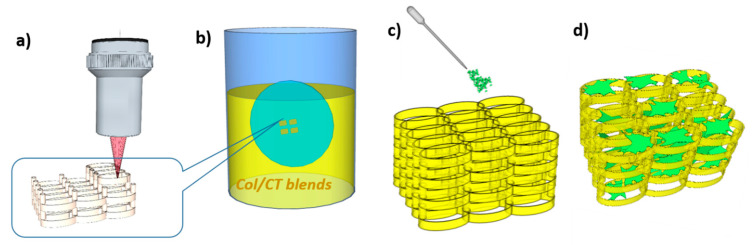
(**a**) Fabrication of complex 3D structures by laser direct writing via two-photons polymerization (LDW via TPP) of IP-L780 photopolymer; (**b**) functionalization of the 3D structures via dip coating in collagen/chitosan (Col/CT) blends; (**c**) osteoblast-like cells seeded on Col/CT functionalized 3D structures; (**d**) biological assays of the cell-seeded on Col/CT functionalized 3D structures.

Figure 5 illustrates the steps followed for structures fabrication, functionalization and in vitro testing of their osteogenic potential.

### 3.1. Materials

The photopolymer (IP-L780) and the developer (PGMEA) used for the fabrication of the 3D structures were purchased from Nanoscribe *GmbH.* IP-L780 is a biocompatible photopolymer designed for LDW via TPP using the Nanoscribe technology. It contains 2-(Hydroxymethyl)-2-[[(1-oxoallyl)oxy]methyl]-1,3-propanediyl diacrylate (>95%) and 7-(Diethylamino)-3-(2-thienylcarbonyl) -2H-1-benzopyran 2-one (<5%). IP-L780 can be easily handled by drop casting (that is as a precursor step for LDW via TPP processing), it has a refractive index at 780 nm (unexposed) of 1.48 and enables to obtain high resolution and mechanical stability for structures in the micro and sub-micron range [13]. Collagen (Col, C9301), Chitosan (CT, 9012-76-4) and acetic acid (45754) used for the functionalization of the 3D structures were purchased from Sigma Aldrich, St. Louis, MO, USA.

### 3.2. Fabrication of 3D Structures

#### 3.2.1. Laser Direct Writing via Two-Photon Polymerization (LDW via TPP) of 3D Structures

The chosen fabrication method for the microstructures is LDW via TPP. This is an emergent 3D printing technology at micrometer scale, capable of reaching as low as 90 nm lateral features. The principal idea of this technology is using two-photon absorption to activate a photoreactive solution (photoinitiator) that in turn determines either chain polymerization processes, or simply breaking molecules into reactive components, all in a confined volume. The light-matter interaction is similar to UV lithographic methods, but the two-photon absorption allows for initiating and controlling the reaction in a very confined volume, at the focal point of the focusing optics. Moreover, this allows for obtaining a laser volume pixel (voxel) significantly smaller than the diffraction limit or beam waist. For microstructure fabrication, we have used a commercially available installation, Nanoscribe Photonic Professional. The laser source is an Er-doped fiber oscillator, followed by an Er-doped fiber amplifier, with a periodically polled LiNbO3 crystal placed at the exit. It delivers 120 fs pulses with a repetition rate of 80 MHz, centered on a wavelength of 780 nm. The laser was focused using a 60x microscope objective. The sample was positioned using a system of XYZ stepper stages and XYZ piezo stages, working concurrently. To eliminate any trace of monomers in the structures, after the laser irradiation process the samples were washed five times in PGMEA developer. The fact that the cells easily grew on all the structures including on the uncoated ones (as shown at Results and Discussion/biological assessments section) certainly proves that there were no remaining monomers in the structures (that would have been highly toxic for the cells).

#### 3.2.2. Functionalization of 3D Structures

Collagen (Col) and Chitosan (CT) were separately dissolved in 0.1 M acetic acid to obtain 1 wt% solutions by ultrasonication for 30 min. The solutions were mixed together in Col/CT blending ratios of 100/0; 80/20/, 60/40, 50/50, 40/60, 20/80, 0/100 by weight. For sonicating the Col, CT and Col/CT solutions, we employed a Sharpertek ultrasonic system (power 780 W, working at 25 °C temperature, frequency 40 kHz provided continuously at intensities of about 60 mW/cm2 SATA (surface averaged time averaged)), for 20 min per solution. The sonication was performed on ice bath and we observed no thermal effects.

For Col/CT functionalization, the 3D structures fabricated by LDW via TPP were manually dipped into Col, CT or Col/CT blends solutions, for 20 s each, at room temperature. Then, each sample was left to dry for one hour, at room temperature.

### 3.3. Characterization of 3D Structures

#### 3.3.1. Morphological Investigations

The morphology of the 3D structures was investigated by Scanning Electron Microscopy (SEM, FEI InspectS model, Hillsboro, OR, USA). Before SEM examination, the structures were coated with a 10 nm thick gold layer.

#### 3.3.2. Wettability

The wetting properties of the materials found in the unfunctionalized i.e., uncoated 3D structures and on the coating materials i.e., Col/CT blends were assessed by water contact angle measurements. The image of the water droplet was captured within 10 s of delivery. All analyses were made at room temperature. A water drop was placed on each investigated surface using a syringe and the contact angle was measured by the goniometer (DSA 10 Control Unit, Krüss, Germany).

### 3.4. Biological Assessments

Ethical Statements: The MG-63 cell line was obtained from European Collection of Cell Cultures (ECACC, Salisbury, UK).

#### 3.4.1. Cell Culture

We used MG-63 osteoblast-like cells from the European Collection of Cell Cultures (ECACC, Salisbury, UK). The cells were cultured in MEM growth medium (Biochrom, Berlin, Germany) supplemented with 10% fetal bovine serum (FBS, Biochrom), 2 mML-glutamine (Biochrom) 1% non-essential amino-acids and 100 IU/mL of penicillin/streptomycin (Biochrom). The cell culturing conditions were standard involving temperature of 37 °C and humidity of 5% CO_2_. Confluent cells were detached from the substrate using 1% Trypsin and then they were seeded on the 3D structures at cell density of 5000 cells/structure. Before cell seeding, the structures were sterilized for two hours using a UV lamp. Next, the cells were cultured for four weeks. In order to avoid the influence of the cells growing around the 3D structures, before preparation for MTS assay, ALP production, Alizarin Red staining and human osteocalcin immunoassay, they were removed using a cell scraper (Trasadingen, Switzerland) under an Axio Imager 2, Zeiss microscope with AxioCam MRm camera.

#### 3.4.2. Morphological Studies on Cells

The cells were cultured on the 3D structures for a time interval of 3 days, under standard conditions. After that, the cells were washed with PBS and fixed with 2.5% glutaraldehyde in PBS during 1 h, at room temperature. Then the cells were washed again and dehydrated in ethanol (EtOH) solution with concentrations of 70%, 90%, respectively 100%. We washed two times for each ethanol concentration, each during 15 min. The resulting samples were immersed in solutions of EtOH-HMDS of 50%:50%; 25%:75%, and 0%:100% ratios. This step was done twice, each time for 3 min. The samples were left to in air and at room temperature.

#### 3.4.3. Cells Viability

The cells were first cultured as described at 3.4.1. The cells were cultured for four weeks. Then the culture medium was replaced with 16.67% MTS (Cell Titer 96^®^ Aqueous One Solution Cell Proliferation Assay, Promega, Madison, WI, USA) and 83.33% MEM (5% FBS). After 3 h of incubation, 100 µL of supernatant from each sample was collected and placed in a 96-well plate. These were analyzed through absorbance measurements that were carried out at 490 nm, using a Mitras LB 940 (Berthold Technologies, BadWildbad, Germany) spectrophotometer. The cells viability was determined as percent from uncoated 3D structures, that were considered the control samples.

#### 3.4.4. ALP Activity

Alkaline Phosphatase activity was quantified using absorbance measurements at a wavelength of 405 nm. For this, we used the Alkaline Phosphatase Assay Kit (Colorimetric) (ab83369) (Abcam, Cambridge, UK). For cell lysate we used p-Nitrophenyl Phosphate Liquid Substrate (pNPP). 40 µL pNPP 5mM liquid standard solution were mixed with 160 µL Assay Buffer. Then, serial dilutions were prepared (0, 4, 8, 12, 16, 20 nmol/well of pNPP). The cells were harvested by trypsinization, washed three times with cold PBS and next resuspended in 100 µL Assay Buffer. To remove the insoluble compounds, the cells were centrifuged at 7000 rpm during 15 min. The supernatant was transferred into 96 well-plates (100 µL/well) and completed with 50 µL of 5mM pNPP solution. We added then 10 µL of ALP enzyme solution into each well. All standards and samples were incubated in the dark, at room temperature, for 60 min. 20 µL of Stop Solution was added into each well. In the end, the absorbance was measured at 405 nm using a Mithras (Berthold Technologies) spectrophotometer. The results were expressed as units per milligram of protein in cell lysate. The protein was assayed by Bradford (B6916, Sigma Aldrich) method. The standard was serum bovine albumin.

#### 3.4.5. Osteocalcin Secretion

For Osteocalcin secretion measurements, the cell-seeded structures were prepared using Quantikine^®^ELISA Human Osteocalcin Immunoassay (Catalog Number DSTCN0 (R&D SYSTEMS, Minneapolis, MN, USA)), according to the producer’s specifications. The standard curve for Osteocalcin calibration was obtained using standard osteocalcin solution in the kit. 50 µL from the supernatant from each sample was added to a 96-well plate, together with 100 µL of Assay Diluent. The samples were incubated while shaking during two hours and washed three times using the washing buffer. Next, 200 µL from the conjugate were added in each well. After two hours of shaking at room temperature, the samples were washed for four times with the washing buffer. Next, 200 µL from the substrate solution was added to each well and incubated during 30 min in the dark. The reaction was finished using 50 µL of the Stop solution. Osteocalcin secretion was measured through the absorbance at wavelength of 450 nm with a correction at 570 nm, using a Mitras LB 940 (Berthold Technologies) spectrophotometer.

#### 3.4.6. Alizarin Red (ARS) Assay

The cells were seeded in a similar protocol as the one used for SEM imaging. After four weeks of incubation, the samples were washed two times with double-distilled water. 1 mL of 40 mM ARS (pH 4.1) was added to each well form a 96-well plate. The samples were incubated for another 20 min, at room temperature and then washed with double-distilled water, during shaking for 5 min. The quantification of the mineralized deposited in the cells seeded on the structures was done by extracting the calcified mineral at low pH, followed by neutralization with ammonium hydroxide and absorbance measurement at 405 nm using a Mitras LB 940 (Berthold Technologies) spectrophotometer.

#### 3.4.7. Statistical Analysis

The experimental data were presented as mean ±STD (standard deviation) of three different experiments in identical conditions. The statistical analysis was performed Student’s test. The data were considered statistically significant for *p* < 0.05. Each data point for the relative cell viability, ALP activity, Alizarin Red assay and osteocalcin secretion was calculated as the mean of the three different measurements performed in three different experiments. In the graphs, the standard deviation was shown as an error bar.

## 4. Conclusions

In this work, we demonstrated a proof of concept for Collagen/Chitosan (Col/CT) functionalization of complex 3D structures that enhanced the osteogenesis in osteoblast-like cell cultures. The structures were fabricated by laser direct writing via two-photons polymerization of IP-L780 biocompatible photopolymer and comprised of hexagonal and ellipsoidal units having the length of 80 µm, width of 40 µm, height of 14 µm and separated by 20 µm cylindrical pillars. Structures’ functionalization was achieved through a simple dip coating process in Col/CT blends with different blending ratios. The morphology and wettability of the functionalized 3D structures showed a strong dependence on the Col/CT blending ratio. Specifically, the water contact angle for Col/CT functionalized structures decreased with increasing CT content in the blends, while the morphology of the structure’s surfaces became smoother. These trends further exerted a direct influence on the cellular attachment on the functionalized structures, which also increased with increasing amount of CT in the blends. The osteogenic role of Col/CT functionalization of the 3D structures was confirmed by the expression of osteogenic markers such as alkaline phosphatase and osteocalcin secretion. In addition, the mineral deposits were evaluated quantitatively through Alizarin Red staining. Both types of structures i.e., having ellipsoidal and hexagonal units, showed similar trends in what concerns the dependence of these osteogenic indicators on the Col/CT blending ratio. Nonetheless, the structures having ellipsoidal units showed better osteogenic performances that those with hexagonal units. For both type of structures, the strongest osteogenic effect was obtained for Col/CT ratio of 20/80, which provided the highest alkaline phosphatase activity, osteocalcin secretion and Alizarin Red staining intensity in the cultured cells as compared to the other samples.

## Supplementary Materials

Supplementary Materials can be found at https://www.mdpi.com/1422-0067/21/17/6426/s1.

## Abbreviations

Col	Collagen
CT	Chitosan
ALP	Alkaline Phosphatase
AR	Alizarin Red
ARS	Alizarin Red Staining
LDW via TPP	Laser Direct Writing via Two-Photon Polymerization
SEM	Scanning Electron Microscopy
PBS	Phosphate Buffered Saline
EtOH	Ethanol
PNPP	P-Nitrophenyl Phosphate
