# Supplementary material for: Collagen/Chitosan Functionalization of Complex 3D Structures Fabricated by Laser Direct Writing via Two-Photon Polymerization for Enhanced Osteogenesis
Source: Int J Mol Sci. 2020 Sep 3;21(17):6426. doi: 10.3390/ijms21176426 (PMC7504713; doi:10.3390/ijms21176426)
Supplement: Supplementary file 1 [file ijms-21-06426-s001.pdf]

Supplementary material

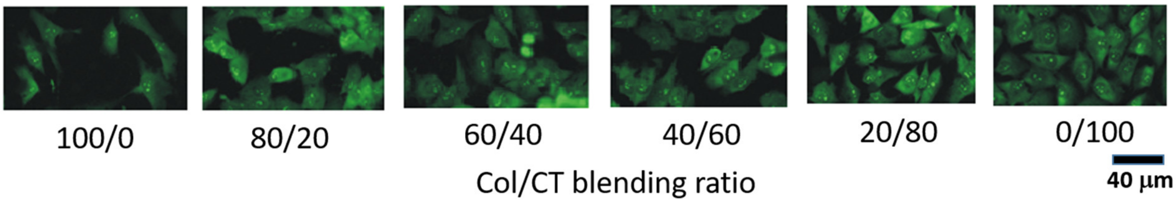

Figure S1 supplementary material Cells attached of flat surfaces of Col/CT with the indicated blending rations, as observed after fluorescent staining with Acridin Orange; to note the cells attachment increasing with increasing CT content in the blends
